# Bacterial Metabolism and Antibiotic Efficacy

**DOI:** 10.1016/j.cmet.2019.06.009

**Published:** 2019-08-06

**Authors:** Jonathan M. Stokes, Allison J. Lopatkin, Michael A. Lobritz, James J. Collins

**Affiliations:** 1 Institute for Medical Engineering & Science, Department of Biological Engineering, and Synthetic Biology Center, Massachusetts Institute of Technology, Cambridge, MA 02139, USA; 2 Infectious Disease & Microbiome Program, Broad Institute of MIT & Harvard, Cambridge, MA 02142, USA; 3 Machine Learning for Pharmaceutical Discovery and Synthesis Consortium, Massachusetts Institute of Technology, Cambridge, MA 02139, USA; 4 Wyss Institute for Biologically Inspired Engineering, Harvard University, Boston, MA 02115, USA; 5 Roche Pharma Research and Early Development, Roche Innovation Center Basel, 4070 Basel, Switzerland; 6 Harvard-MIT Program in Health Sciences and Technology, Cambridge, MA 02139, USA

**Keywords:** bacterial metabolism, antibiotic mechanism, antibiotic tolerance, antibiotic adjuvants

## Abstract

Antibiotics target energy-consuming processes. As such, perturbations to bacterial metabolic homeostasis are significant consequences of treatment. Here, we describe three postulates that collectively define antibiotic efficacy in the context of bacterial metabolism: (1) antibiotics alter the metabolic state of bacteria, which contributes to the resulting death or stasis; (2) the metabolic state of bacteria influences their susceptibility to antibiotics; and (3) antibiotic efficacy can be enhanced by altering the metabolic state of bacteria. Altogether, we aim to emphasize the close relationship between bacterial metabolism and antibiotic efficacy as well as propose areas of exploration to develop novel antibiotics that optimally exploit bacterial metabolic networks.

## Teaser

The metabolic state of bacteria significantly contributes to the efficacy of antibiotics. In this Perspective, Stokes et al. highlight the close relationship between bacterial cell metabolism and antibiotic efficacy, leveraging prior observations to describe areas for further exploration, with the goal of developing next-generation antibiotics that can optimally exploit the complex metabolic networks of bacteria.

## Main Text

### Introduction

Since their clinical implementation 8 decades ago, antibiotics have become the foundation of modern medicine. However, their continued efficacy is threatened by the global dissemination of antibiotic-resistance determinants, driven in large part by improper use of antibiotics in clinical, community, and agricultural settings (Bush et al., 2011). To develop effective next-generation antibacterial therapies, it is imperative that we gain a more thorough understanding of how bacteria respond to antibiotics and leverage this understanding toward the development of treatments that expand drug efficacy beyond the current state of the art.

**Figure 1:**
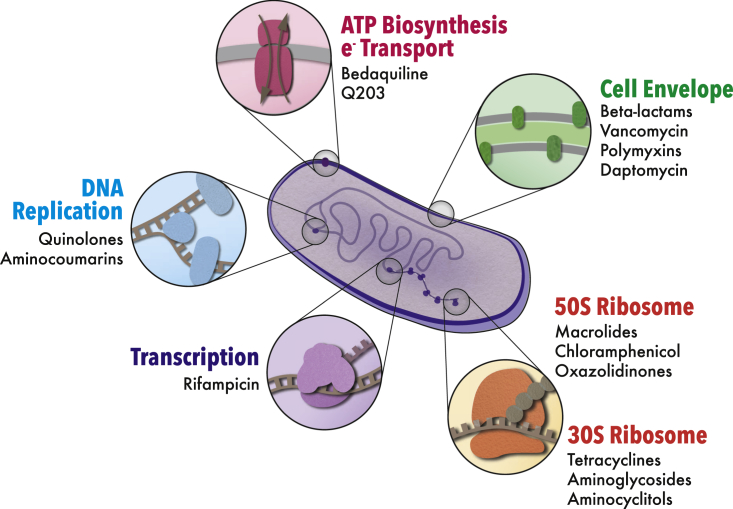
Cellular Processes Targeted by Conventional Antibiotics Although a relatively large number of clinically available antibiotics have been developed, these collectively target a narrow spectrum of macromolecular biosynthetic processes. With few exceptions, target processes can be grouped into four primary categories: cell envelope biogenesis, DNA replication, transcription, and protein biosynthesis.

Our modern antibiotic arsenal has largely resulted from screens designed to identify molecules that inhibit bacterial growth *in vitro* (Brown and Wright, 2016). Despite an impressive number of individual bioactive compounds discovered through this approach—on the order of hundreds—only a handful of cellular processes are targeted (Kohanski et al., 2010). With a few exceptions, these can be grouped into (1) cell envelope biogenesis, (2) DNA replication, (3) transcription, and (4) protein biosynthesis (Figure 1). Given their roles in facilitating cell growth and division, these processes collectively consume the major fraction of the metabolic output of the cell, with protein biosynthesis alone accounting for upward of 70% of ATP utilization (Stouthamer, 1973). Thus, it is not surprising that the perturbation of these energy-consuming processes by antibiotics induces significant, yet frequently overlooked, perturbations to metabolic homeostasis (Belenky et al., 2015, Zampieri et al., 2017).

In this perspective, we emphasize the importance of these metabolic consequences of antibiotic treatment by describing three postulates that define antibiotic efficacy in the context of bacterial metabolism:

1. Antibiotics alter the metabolic state of bacteria, which contributes to the resulting death or stasis;
2. The metabolic state of bacteria influences their susceptibility to antibiotics; and
3. Antibiotic efficacy can be enhanced by altering the metabolic state of bacteria.

We believe that these postulates unify decades of independent observations into a mechanistically coherent framework, which will allow for the more rational development of antibiotics and synergistic therapeutic combinations going forward. Overall, we aim to highlight past successes and propose areas for further exploration toward the development of next-generation antibiotics that exploit the expansive and complex network that defines bacterial metabolism.

### Antibiotics Alter the Metabolic State of Bacteria, which Contributes to the Resulting Death or Stasis

The positive relationship between bacterial growth rate and bactericidal antibiotic efficacy has been known for decades (Eng et al., 1991, Lee et al., 2018, Tuomanen et al., 1986). The implications of this relationship are epitomized by current *Mycobacterium tuberculosis* treatment regimens—month-long courses of therapy are required for successful eradication of infection due to the high frequency of slow-growing or non-growing cells (Gill et al., 2009, Gomez and McKinney, 2004, Muñoz-Elías et al., 2005). However, beyond the conventional metric of growth rate, investigators studying *M. tuberculosis* have come to appreciate that a direct relationship exists between bacterial metabolism and bactericidal antibiotic efficacy (Baek et al., 2011, Bald et al., 2017). This subtlety has been largely overlooked by researchers studying more rapidly growing model organisms such as *Escherichia coli*, in part due to the fact that their metabolic states have not until recently been considered barriers for the efficient and effective treatment of infection.

Given that antibiotic development against *M. tuberculosis* demands that investigators put bacterial metabolism at the forefront, it is not surprising that studies in this organism have revealed important insights into the antibiotic-induced metabolic dysregulations that contribute to bacterial cell death or stasis. For example, one simple observation that embodies this notion is that the efficacy of bactericidal antibiotics against *M. tuberculosis* is highly dependent on the concentration of dissolved oxygen in the environment. Indeed, prior work has revealed that increased oxygen tension enhances bactericidal antibiotic efficacy against *M. tuberculosis* by permitting elevated oxidative damage to cellular macromolecules as a result of antibiotic exposure (Grant et al., 2012). Moreover, chemically regulating the intracellular accumulation of promiscuously reactive free radical species using the hydroxyl radical scavenger thiourea can modulate the killing of *M. tuberculosis* by controlling the extent of oxidative damage that occurs as a downstream metabolic byproduct of bactericidal antibiotics (Grant et al., 2012, Pandey and Rodriguez, 2012).

Recent studies have expanded on these observations by showing more precisely that antibiotic-induced oxidation of deoxycytidine triphosphate (dCTP) pools in mycobacteria contributes to bactericidal drug efficacy. Interestingly, dCTP oxidation is observed in cells treated not only with DNA replication inhibitors but also with rifampicin and streptomycin, which do not inhibit DNA replication machinery as a primary target (Fan et al., 2018). Furthermore, the delayed bactericidal response induced by the F_1_F_0_ ATP synthase inhibitor bedaquiline has been shown to be the result of an extensive metabolome remodeling that attempts to compensate for intracellular ATP depletion (Koul et al., 2014). Together, these data support that a series of metabolically driven molecular events contribute to the activity of functionally diverse bactericidal antibiotics.

Unsurprisingly, the fact that antibiotics induce downstream metabolic perturbations as intrinsic facets of their mechanism is not limited to *M. tuberculosis*. Indeed, in the more rapidly growing model organisms *E. coli* and *Staphylococcus aureus*, it has similarly been shown that functionally diverse bactericidal antibiotics of the β-lactam, quinolone, and aminoglycoside classes induce the production of highly reactive free radicals, which promiscuously react with and damage intracellular macromolecules, contributing to death (Dwyer et al., 2014, Dwyer et al., 2007, Foti et al., 2012, Kohanski et al., 2007, Vatansever et al., 2013). Moreover, this oxidative stress was shown to coincide with a remodeling of the metabolome characterized by an increase in abundance of central carbon metabolites and a decrease in concentrations of free lipids and nucleotide pools (Belenky et al., 2015). These data support the notion that antibiotic-induced metabolic perturbations are physiologically diverse and that the observation of free radicals is just one manifestation of the global metabolic consequences of treatment. Furthermore, detailed studies measuring bacterial respiration revealed that *E. coli* and *S. aureus* treated with bactericidal antibiotics display increased respiratory activity and that increasing basal respiration rates via deletion of the alpha subunit of the F_1_ complex of ATP synthase (*atpA*) increases bactericidal antibiotic efficacy relative to wild-type cells (Lobritz et al., 2015).

**Figure 2:**
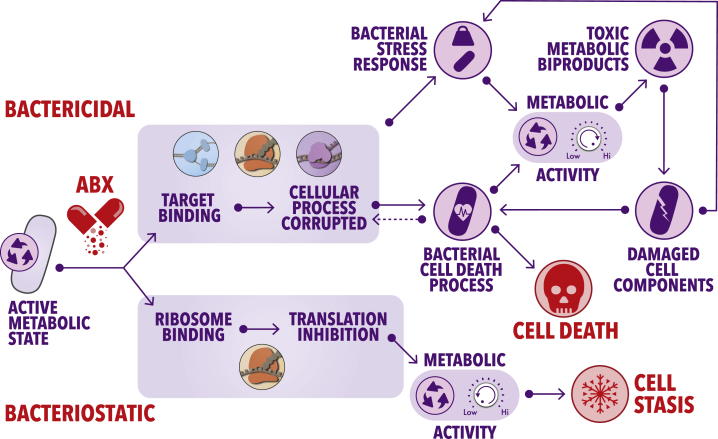
Models of the Metabolic Consequences of Treatment with Bactericidal and Bacteriostatic Antibiotics Primary target corruption by bactericidal antibiotics causes damage to essential macromolecules within the cell. This leads to the induction of stress response pathways to alleviate the deleterious consequences of the initial target corruption, which increases metabolic activity to meet the corresponding energy demands. The heightened metabolic output results in the production of toxic metabolic byproducts such as reactive species, which promiscuously damage macromolecules, leading to the induction of additional stress response pathways, thus once again increasing metabolic load. This process continues until the cycle terminates with bacterial cell death. Bacteriostatic antibiotics, on the other hand, tend to strictly inhibit protein biosynthesis (or transcription in certain contexts). This leads to a decrease in metabolic activity and subsequent cell stasis.

Altogether, these observations are consistent with a model where primary target corruption by bactericidal antibiotics results in collateral damage to intracellular macromolecules, inducing a cycle of elevated stress responses and concurrently increased metabolic activity, which terminates with cell death (Figure 2). Importantly, this generalized model is consistent with seminal work by Cho et al., which revealed that the β-lactam antibiotic mecillinam induces an energy-demanding futile cycle of cell wall biosynthesis and degradation that depletes cellular resources (Cho et al., 2014), thereby increasing the metabolic rate that contributes to lethality. Similarly, it has been shown that genetically inducing ATP-consuming futile cycles in *E. coli* through the expression of *pck*, *acs*, and *atpAGD* significantly sensitizes cells to killing by exogenous oxidative stress (Adolfsen and Brynildsen, 2015). Recent work has additionally shown that bactericidal antibiotics of wide-ranging functions continue to induce reactive oxygen species accumulation and death of *E. coli* even after their removal from cells (Hong et al., 2019). While the precise mechanism underlying this self-amplifying accumulation of reactive molecules remains to be determined, ongoing work will likely shed light on the energy-dependent cycles that increase metabolic activity and macromolecule damage, which contribute to bacterial cell killing upon exposure to bactericidal compounds.

In contrast to bactericidal antibiotics, work with bacteriostatic agents has revealed that these molecules induce largely opposing effects on bacterial metabolism (Lin et al., 2014). Treatment of *E. coli* with an array of bacteriostatic translation inhibitors has been shown to cause decreased cellular respiration in a manner similar to genetically disrupting cytochrome oxidase production (Lobritz et al., 2015). Furthermore, *S. aureus* treated with chloramphenicol displays a metabolic profile in which amino acid, ATP, and NADH abundances increase, consistent with diminished energy utilization and reduced macromolecule biosynthesis (Figure 2).

Interestingly, when bactericidal antibiotics are combined with bacteriostatics, the phenotypic outcome is dominated by the latter (Brown and Alford, 1984, Johansen et al., 2000, Rocco and Overturf, 1982, Weeks et al., 1981, Winslow et al., 1983). An exemplary case is that chloramphenicol has a dominant effect over the bactericidal action of quinolones in *E. coli*, resulting in stasis rather than death (Lobritz et al., 2015). Furthermore, recent work in *M. tuberculosis* has shown that chemical inhibition of the F_1_F_0_ ATP synthase by sub-inhibitory concentrations of bedaquiline, or of the terminal respiratory oxidase cyt-*bc*_*1*_*:aa*_*3*_ by Q203, is dominantly protective over the bactericidal actions of isoniazid and moxifloxacin (Lee et al., 2019). In these cases, it is hypothesized that the dampened metabolic state induced by bacteriostatic drug treatment prevents the activation of those downstream metabolic cycles commonly induced by bactericidal drugs, which result in widespread damage to cellular macromolecules Together, these data emphasize the biological significance of the metabolic perturbations resulting from antibiotic treatment—whether bactericidal or bacteriostatic—and support the notion that metabolic perturbations far removed from primary target corruption contribute to the phenotypic outcome of treatment (Figure 2).

### The Metabolic State of Bacteria Influences Their Susceptibility to Antibiotics

Given that antibiotic treatment significantly alters the metabolic state of bacteria, it follows that the metabolic state of bacteria also influences their intrinsic susceptibility to the deleterious effects of antibiotics. Indeed, as mentioned above, genetically increasing the basal respiration rate of *E. coli* increases bactericidal antibiotic efficacy over wild-type cells (Lobritz et al., 2015). The opposite is also true; consider stationary phase growth as a familiar physiologic state characterized by resource exhaustion, repressed metabolic activity, and tolerance to bactericidal antibiotics (Kolter et al., 1993, Navarro Llorens et al., 2010). *E. coli* has been observed to display a time-dependent decrease in ATP concentration upon transition from log phase to stationary phase (Schneider and Gourse, 2004). Moreover, high-resolution kinetic analysis of metabolite dynamics in carbon-starved *E. coli* revealed that such cells display a significant decrease in the abundance of succinate and reduced glutathione relative to glucose-supplemented cultures (Link et al., 2015). Predictably, these data are consistent with a metabolic downshift coinciding with nutrient deprivation.

Based on previously discussed observations that bacteriostatic antibiotics antagonize bacterial cell killing by bactericidal drugs, one may expect to observe similar metabolic signatures between bacteriostatic-treated cells and those in stationary phase. This is true to an extent. However, when analyzing the proteome and metabolome of cells treated with bacteriostatic antibiotics, somewhat discrete patterns do emerge. While previous work revealed, expectedly, that treatment of cells with bacteriostatic compounds decreases cellular respiration (Lobritz et al., 2015) and downregulates glycolysis and gluconeogenesis, pyruvate metabolism, and the tricarboxylic acid (TCA) cycle (Lin et al., 2014), it was concurrently observed that the bacteriostatic antibiotics chloramphenicol and linezolid caused accumulation of ATP, ADP, and AMP as well as NADH and central carbon metabolites (Lobritz et al., 2015).

These data raise intriguing questions: how can bacteriostatic antibiotics simultaneously decrease central carbon metabolism and increase the abundance of associated intracellular metabolites, and how does this relate to bactericidal antibiotic efficacy? The answers to these questions might lie in global metabolic flux. Resource depletion—such as that observed in stationary phase—results in decreased metabolic rates and decreased intracellular concentrations of high-energy metabolites because cells lack access to nutrients required to generate a sufficient adenylate charge to drive energy-consuming biosynthetic processes. The result is a lack of global metabolic flux that limits bactericidal antibiotic efficacy. On the other hand, treatment with bacteriostatic antibiotics decreases metabolic flux through not only the processes that are immediately inhibited by these drugs (mainly protein biosynthesis) but biologically distant functions as well, thereby causing in an increase of intracellular metabolites that are left unused. Indeed, chloramphenicol treatment results in decreased metabolic activity and the accumulation of amino acids, nucleotides, and lipids, as does rifampicin (Lobritz et al., 2015). This is consistent with inhibition of processes that directly consume these metabolites (amino acids and nucleotides), in addition to cell cycle progression as a downstream consequence (in the case of lipids). Importantly, in both cases—stationary phase and bacteriostatic-treated cells—metabolic flux is diminished, resulting in decreased efficacy of bactericidal antibiotics that rely on downstream metabolic processes to eradicate cells. The absolute abundances of intracellular metabolites that accompany these repressed metabolic rates appear to be a consequence of the mechanism underlying the metabolic suppression and not a factor that necessarily governs bactericidal antibiotic efficacy.

Importantly, the notion that the metabolic state of bacteria influences their susceptibility to antibiotics can be further extended to other metabolically repressed conditions that are associated with a loss of bactericidal drug efficacy. For example, bacterial cells embedded in biofilms are notoriously difficult to eradicate due to a combination of drug impermeability through the biofilm matrix, as well as decreased metabolic activity of the cells within (Høiby et al., 2010a, Stewart and Franklin, 2008). Indeed, as one might expect, high-density biofilms display steep gradients of nutrient and oxygen availability from the periphery of the biofilm to the center, resulting in metabolic dormancy of the majority of the community located toward its interior. Notably, this is somewhat similar to stationary phase cultures in liquid medium discussed earlier. Such metabolically repressed antibiotic-tolerant cells residing in biofilms are commonly observed in the model biofilm-producing organism *Pseudomonas aeruginosa in vitro*, as well as from the sputum of cystic fibrosis patients (Høiby et al., 2010b), emphasizing the importance of metabolic repression that underlies the loss of bactericidal drug efficacy against cells in such communities.

Furthermore, persister cells are a subpopulation of bacteria within a larger antibiotic-susceptible population that display decreased susceptibility to bactericidal agents through mechanisms involving metabolic repression (Brauner et al., 2016). While the molecular mechanisms that result in the emergence of persister subpopulations in growing cultures are diverse and remain poorly understood, one striking commonality is that these cells universally display restricted metabolic potential (Prax and Bertram, 2014). For instance, in *S. Aureus*, it has been shown that persisters are produced due to a stochastic entrance into stationary phase by a subpopulation of cells in an active culture, which is accompanied by a decrease in intracellular ATP concentration and upregulation of genes commonly expressed in stationary phase (Conlon et al., 2016). Other investigations have revealed similar phenotypes in *E. coli* and *M. tuberculosis* (Gurnev et al., 2012, Shan et al., 2017). Together, although entry stationary phase, treatment with bacteriostatic drugs, biofilm formation, and persister development are seemingly discrete mechanisms that result in decreased efficacy of bactericidal antibiotics, these investigations in their totality provide strong support that a lack of global metabolic activity is the underlying physiologic driver of this stark phenotypic convergence.

### Antibiotic Efficacy Can Be Enhanced by Altering the Metabolic State of Bacteria

**Figure 3:**
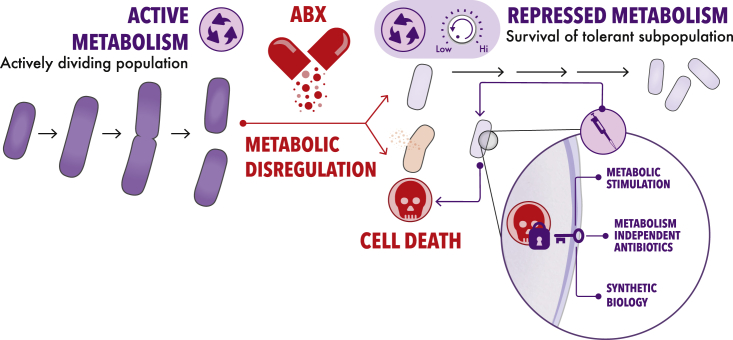
Repression Displayed by Antibiotic-Tolerant Bacteria Can Be Overcome through Multiple Emerging Approaches Antibiotic-tolerant bacteria—whether nutrient-limited stationary phase cells, persisters, or biofilms—all display repressed metabolism that contributes to their ability to survive bactericidal antibiotic treatment. Metabolic activation through the use of metabolite adjuvants has been shown to enhance the sensitivity of antibiotic-tolerant bacteria to conventional bactericidal antibiotics. Furthermore, molecules like polymyxins, human cationic peptides, and ADEP4 display bactericidal activity that is independent of the metabolic state of the cell and may represent a promising approach to develop antibiotics that are not impeded by metabolic repression displayed by conventionally antibiotic-tolerant populations. Lastly, engineered phage and bacteria that actively modulate the metabolic response of pathogens to conventional antibiotics may be an alternative approach to eradicate cells in metabolically repressed states.

Since the metabolic state of bacteria is a unifying feature defining antibiotic efficacy across a wide range of physiologic states, this greatly simplifies the search for mechanisms through which bactericidal antibiotic activity can be enhanced. Indeed, rather than necessitating the identification of functionally unique adjuvant methods for each specific physiologic state in which a bacterium may exist, modulating metabolic activity is a generalizable approach to enhance bactericidal drug-dependent killing (Figure 3).

Such a strategy was first implemented nearly a decade ago to improve the efficacy of aminoglycosides against antibiotic-tolerant *E. coli* and *S. aureus* populations (Allison et al., 2011). In this study, the authors showed that gentamicin in combination with metabolites found in upper glycolysis (glucose, mannitol, and fructose) could kill *E. coli* persister cells ∼3 logs greater than gentamicin alone. It was found that these metabolites increased the proton-motive force via activation of the electron transport chain, thereby enhancing aminoglycoside uptake and increasing intracellular concentrations of the antibiotic. Indeed, protein biosynthesis occurs even in metabolically repressed states (Gefen et al., 2014), suggesting that the aminoglycosides were bactericidal through their canonical mechanism of action. In a similar manner, more contemporary work has applied a comparable metabolite potentiation strategy against stationary phase cultures of *P. aeruginosa*, where these antibiotic-tolerant cells were killed by tobramycin in combination with a variety of TCA cycle intermediates (Meylan et al., 2017).

Remarkably, this strategy of metabolite-dependent aminoglycoside potentiation has also been applied to aminoglycoside-resistant bacteria (Peng et al., 2015). After observing that spontaneous kanamycin-resistant *Edwardsiella tarda* LTB4 cells were deficient in alanine and glucose relative to their wild-type parent, Peng et al. showed that supplementation of either (or both) of these metabolites in combination with kanamycin resulted in significantly enhanced bactericidal activity. Subsequent investigations revealed that this was through the same mechanism as described above, namely increased kanamycin uptake by TCA cycle activation and proton-motive force enhancement. Here, it is likely that the elevated intracellular concentration of kanamycin overcame the resistance limit of the spontaneous suppressor mutations, thereby resulting in sufficient mistranslation rates to induce death. Importantly, it should be noted that this approach may be ineffective against alternative mechanisms of resistance, such as the acquisition of horizontally acquired aminoglycoside modifying enzymes. Indeed, since antibiotic-resistance determinants can significantly impact wide-ranging aspects of bacterial cell metabolism (Martínez and Rojo, 2011), more thorough studies will be required to determine the limitations of this general approach against bacteria harboring discrete functional classes of antibiotic-resistance determinants.

In addition to metabolite-aminoglycoside synergy against conventionally antibiotic-tolerant cells, unique approaches have also been explored to enhance the activity of quinolone antibiotics against metabolically repressed populations. For example, recent work revealed that stationary phase cultures of *E. coli*, *S. aureus*, and *Mycobacterium smegmatis* can be killed by fluoroquinolones in combination with glucose and a suitable terminal electron acceptor such as fumarate or molecular oxygen (Gutierrez et al., 2017, Gutierrez et al., 2018). Here, the efficacy of quinolones in combination with glucose and an electron acceptor suggests that stationary phase tolerance to this class of antibiotics, in the absence of metabolite adjuvants, is due to impairments in respiratory metabolism. Indeed, in this case, quinolone uptake into cells was shown not to be impacted by the presence or absence of metabolic adjuvants.

Excitingly, recent work has begun to expand on these previous efforts by applying machine-learning approaches to identify metabolites that can modulate the efficacy of diverse bactericidal antibiotics against *E. coli* (Yang et al., 2019). Here, using a combination of phenotypic screening, metabolic network modeling, and white-box machine learning, it was shown that intracellular adenine limitation contributed to the efficacy of ampicillin, ciprofloxacin, and gentamicin. Indeed, amongst numerous additional observations, supplementation of growth media with adenine resulted in decreased killing of *E. coli* by these antibiotics, suggesting that the limitation of adenine by antibiotic treatment stimulates purine biosynthesis, thereby increasing ATP demand and resulting in an enhanced metabolic rate that contributes to cell death (Figure 2). Using their model, the authors additionally showed that pyrimidine supplementation, particularly uracil, could enhance antibiotic lethality. Here, elevated pyrimidine concentrations in the growth medium were postulated to decrease *de novo* pyrimidine biosynthesis and therefore promote purine biosynthesis through the accumulation of 5-phospho-α-D-ribose 1-diphosphate (PRPP).

In summary, the work described above embodies the fact that metabolic modulation via exogenous supplementation of specific metabolites is capable of potentiating conventional antibiotics against bacteria in a wide variety of metabolic states. Moreover, emerging evidence suggests that metabolic modulation can overcome antibiotic tolerance, as well as *bona fide* resistance. As such, the field is ripe for the continued development of additional unique approaches to increase the capabilities of antibiotics.

### Future Outlook

In the preceding sections, we outlined three postulates that define antibiotic efficacy in the context of bacterial metabolism. We first explored how bactericidal and bacteriostatic antibiotics differentially alter the metabolic state of bacteria and how these metabolic consequences contribute to the resulting cell death or stasis. Next, we described how the metabolic state of bacteria influences their intrinsic susceptibility to antibiotics. Lastly, we provided contemporary examples of how exogenous manipulation of the metabolic state of bacteria can increase the efficacy of antibiotics against both antibiotic-sensitive and antibiotic-resistant cells. We would like to note here that despite initial conflicting reports on the role of metabolic processes in contributing to lethality (Keren et al., 2013, Liu and Imlay, 2013), the abundance of unconnected yet supporting studies across an array of organisms, experimental designs, physiological states, and infection models has led to an increasingly convergent and widely established basis in support of the model proposed herein (reviewed in Dwyer et al. [2015]).

The observations discussed throughout this perspective have illuminated multiple paths forward toward the development of next-generation antibiotics that more completely exploit the complex metabolic networks of bacteria. First, we envision that continued applications of genome-scale metabolic network modeling (Feist and Palsson, 2008, McCloskey et al., 2013, Michener et al., 2012, Nudler and Mironov, 2004) coupled with machine-learning approaches (Camacho et al., 2018, Yang et al., 2019) that incorporate multi-scale data—including proteomic, transcriptomic, and metabolomic datasets—will enable systems-level investigations into additional metabolic processes that play important roles in the cellular response to antibiotic exposure. When gathered in a manner that incorporates the temporal dynamics of bacterial responses to antibiotic treatment, this approach has high potential to identify unexplored metabolic adjuvants that are far removed from primary target inhibition, both physiologically and temporally. Moreover, the opportunity exists that metabolic modulation may endow conventionally bacteriostatic drugs with bactericidal capabilities. We emphasize here that these machine-learning-driven approaches should be applied to both conventional and unconventional growth conditions, particularly those that more closely mimic the chemistry found at infection sites. This may result in the identification of metabolic adjuvants that are highly specific for certain infection environments (Cornforth et al., 2018). Indeed, emerging work is revealing that the addition of sodium bicarbonate—the pH buffer in human tissues—to conventional bacterial growth media significantly alters the potency of many classes of conventional antibiotics

(Farha et al., 2018), suggesting that infection-site-specific adjuvants may be possible.

Complementary to the application of large-scale multi-omics and machine-learning approaches, increased studies into the genetic contributors of antibiotic susceptibility in unconventional metabolic states has significant potential to reveal unexplored adjuvant targets. For example, recent work by Stokes et al. revealed genes, which when deleted, sensitized stationary phase colonies of *E. coli* to ciprofloxacin (Stokes et al., 2019). Interestingly, those gene-deletion mutants that were sensitive to killing by ciprofloxacin in the context of stationary phase colonies were distinct relative to those that displayed sensitivity to ciprofloxacin under conventional growth inhibition conditions. Indeed, rather than observing ciprofloxacin hypersensitivity in strains that are deficient in DNA-damage repair, as is seen in metabolically replete conditions, strains that were sensitive to killing in stationary phase were enriched for functions in cell envelope biosynthesis, as well as nucleotide and carbohydrate metabolism. As such, it is likely that continued exploration into antibiotic adjuvant targets against cells in both active and repressed metabolic states will reveal context-dependent methods of eradication, thereby further increasing the breadth of antibiotic potentiation methods.

Beyond identifying methods to rationally and precisely modulate bacterial metabolism to enhance the efficacy of antibiotics, an orthogonal approach that may be advantageous involves subverting metabolic dependence altogether. In particular, bacterial cell death mediated by physical disruption of the cell envelope, as is seen with molecules like polymyxins and cationic peptides, are not largely reliant on the metabolic state of the cell (Cui et al., 2016) and offer mechanistic principles on which to design novel antibiotics to target bacteria irrespective of their metabolic state. Importantly, a plethora of common antiseptics display this mechanism of action (McDonnell, and Russell, 1999); however, these have poor specificity for bacterial membranes and thus result in significant human toxicity. Therefore, future endeavors to identify metabolism-independent bactericidal molecules must be designed such that bacterial cell specificity is paramount. Excitingly, one such example that may serve as a promising proof of concept is ADEP4, a compound that can eradicate *S. aureus* persisters by activating the ClpP protease in an ATP-independent manner (Conlon et al., 2013).

Where population-level investigations may provide novel functional and therapeutic insight into mechanisms through which conventional antibiotic efficacy can be enhanced via metabolic modulation, so too might studies into bacterial population heterogeneity. Indeed, it was recently shown that ciprofloxacin-induced mutagenesis is a phenomenon that occurs in a subpopulation of bacteria, rather than stochastically across an entire culture (Pribis et al., 2019). Specifically, in this study sub-inhibitory concentrations of ciprofloxacin were shown to induce DNA breaks and activate the SOS response in all *E. coli* cells in culture. However, mutagenesis was observed to be limited to a subpopulation in which elevated electron transfer together with the SOS response induced the production of reactive oxygen species. This in turn activated the σ^S^ general stress response, which promoted mutagenic DNA-damage repair. Importantly, this suggests that a metabolically heightened subpopulations are dominantly responsible for the evolution of resistance, which provides important insight into future avenues to modulate metabolism such that resistance evolution and bacterial cell lethality are optimally balanced.

Finally, as parallel fields progress, an exciting opportunity exists in utilizing synthetic biology strategies as therapeutic deliverables. Such an approach could involve using horizontally mediated gene transfer as a mechanism for delivering genetic effectors that serve as metabolic modulators (Lu and Collins, 2009). Furthermore, the engineering of complex microbial consortia with defined properties is becoming increasingly practical. Native microbial communities are particularly advantageous, since the human commensal microbiota serves as a primary line of defense against invading microbes. This phenomenon, known as colonization resistance, is already being leveraged against *Clostridioides difficile* infections through fecal microbiota transplantation (Beaugerie and Petit, 2004, Rea et al., 2011). The precise mechanisms of protection are not fully understood; however, this example offers a promising avenue to pursue that capitalizes on natural microbial interactions as adjuvants in a manner that is specific to anatomical locations (Buffie and Pamer, 2013). Indeed, since metabolic cross-talk commonly occurs between species in native communities, such networks (Camacho et al., 2018), once understood, can be leveraged as a way to alter the metabolic state of pathogenic microbes *in situ* while maintaining the health of the native microbiota (Sung et al., 2017). In this manner, engineered consortia could serve as living adjuvants. This is a particularly exciting frontier, since unlike small molecules, engineered bacterial populations have the capacity to sense and respond to stimuli in real time, and may evolve to combat concurrently evolving pathogens (Brenner et al., 2008, Mao et al., 2018, Saeidi et al., 2011).

We are in the infancy of understanding bacterial metabolism in the context of antibiotic efficacy. As such, we suggest that a transdisciplinary approach including biology, chemistry, physics, and engineering is essential to improve bacterial infection therapy beyond the current state of the art. With a focused effort, we posit that a thorough understanding of the relationship between bacterial cell metabolism and antibiotic function can soon be leveraged into highly potent and precise antibacterial therapies that overcome many of the defense mechanisms used by bacteria to deplete the efficacy of our current antibiotics.
